# Evaluation of warning strategies for paraneoplastic neurological syndromes associated with PD-1/PD-L1 inhibitors

**DOI:** 10.3389/fimmu.2025.1670751

**Published:** 2025-12-18

**Authors:** Zhuangzhuang Ren, Yudan Liu, Jianguo Liu, Xiaokun Qi, Feng Qiu, Chenjing Sun

**Affiliations:** Senior Department of Neurology, Chinese PLA Hospital, Beijing, China

**Keywords:** autoantibodies, immune checkpoint inhibitors, immunotherapy, neurotoxicity, paraneoplastic neurological syndromes

## Abstract

**Background:**

The suppressive effects of immune checkpoint inhibitors (ICIs) on anti-tumor immunity have been well documented. However, ICIs can enhance immune responses and trigger autoimmune-related diseases by blocking PD-1 or PD-L1. The worst prognosis is observed in paraneoplastic neurological syndromes (PNS). This study aimed to evaluate the clinical characteristics of PD-1/PD-L1 inhibitor–related PNS and the prognostic impact of antibody subtypes, with the goal of enabling pre-treatment risk warning.

**Methods:**

This was a retrospective descriptive study involving 224 patients with PD-1/PD-L1 inhibitor–related PNS from May 2015 to May 2025, including 8 patients who presented at our hospital and 216 patients reported in the literature. According to the July 2021 international consensus diagnostic framework for PNS, patients were stratified into risk-antibody (high-, intermediate-, and low-risk), unknown-risk antibody, and antibody-negative groups. Clinical features, primary tumor type, ICI regimen, autoantibody profile, treatments, and outcomes were analyzed. Risk-antibody subtypes were further explored.

**Results:**

There were 112 patients in the risk-antibody group (87 high-risk, 20 intermediate-risk, and 5 low-risk), 51 in the unknown-risk antibody group, and 61 in the antibody-negative group. The risk-antibody group showed a higher incidence of limbic encephalitis, subacute cerebellar degeneration, and subacute sensory neuronopathy. The prognosis was worse in the risk-antibody group, with a mortality rate of 29%, significantly higher than 17% in the unknown-risk group and 10% in the antibody-negative group (P = 0.012). Anti-Hu–positive patients were mainly diagnosed with limbic encephalitis, encephalomyelitis, and subacute cerebellar degeneration, with a mortality rate of 23%. Anti-Ma–positive patients primarily presented with encephalomyelitis, limbic encephalitis, and subacute cerebellar degeneration, with a mortality rate of 35%. Anti-Yo–positive patients were mainly associated with subacute cerebellar degeneration, with a mortality rate of 25%. The mortality rate among Anti-amphiphysin–positive patients was 33%. In contrast, 71% of Anti-NMDAR–positive patients had favorable outcomes.

**Conclusion:**

Among patients with PD-1/PD-L1 inhibitor–related PNS, those with risk-antibody positivity had worse prognoses, while patients with unknown-risk antibodies had outcomes similar to those with antibody negativity, suggesting that unknown-risk antibodies are not directly pathogenic or may elicit weaker immune responses. Pre-treatment screening for PNS-related antibodies is recommended, as it may facilitate early warning, identify high-risk patients, and help prevent autoimmune-related diseases caused by excessive immune modulation. After disease onset, efficient immunomodulatory treatment tailored to antibody subtypes may improve outcomes in risk-antibody–positive patients.

## 1 Introduction

Immune checkpoints are negative regulatory molecules in the immune system, such as cytotoxic T-lymphocyte–associated antigen 4 (CTLA-4), programmed cell death protein 1 (PD-1), and Programmed Death-Ligand 1 (PD-L1). Cancer cells can upregulate PD-L1 under the influence of interferon-gamma (IFN-γ), delivering inhibitory signals to T cells and thereby evading anti-tumor immunity. PD-1 inhibitors block PD-1 receptors on host immune cells, whereas PD-L1 inhibitors block PD-L1 expressed on certain tumor cells, thus unleashing anti-tumor immune responses and producing anticancer effects (1, 2). Although PD-1/PD-L1 inhibitors can have significant therapeutic effects, they may also lead to excessive immune responses, potentially inducing autoimmune diseases (3, 4), with an increasing incidence of immune checkpoint inhibitor-related neurological adverse events (n-irAEs) (5), including encephalitis, meningitis, Guillain-Barré syndrome, myasthenia gravis-like syndrome, various demyelinating syndromes, as well as sensory and motor neuropathy (6). The incidence of ICI monotherapy is approximately 4.2%, while it increases to around 14% with CTLA-4 combination therapy (7).

Paraneoplastic neurological syndromes (PNS) are cancer-associated neurological disorders mediated by autoimmune mechanisms and autoantibodies directed against neuronal epitopes (8). Their complex pathogenesis may involve soluble factors such as tumor-secreted hormones or cytokines, or immune responses targeting tumor cells that cross-react with self-antigens (9–11). For PD-1/PD-L1 inhibitor–related PNS, current treatment generally consists of discontinuing immune checkpoint inhibitors (ICIs), while adopting individualized immunomodulatory regimens including corticosteroids, intravenous immunoglobulin, plasma exchange, anti–tumor necrosis factor-α antibodies, tacrolimus, methotrexate, cyclophosphamide, interleukin-17 inhibitors, or rituximab (12, 13). Recent reviews have shown that some severe n-irAEs are poorly responsive to corticosteroids and may require early escalation to plasma exchange, IVIG, or other targeted immunomodulatory treatments (14). Multicenter studies further indicate that n-irAEs can follow acute, monophasic, or chronic courses, with neurological deficits frequently persisting beyond twelve weeks. In the ICI setting, applying updated consensus criteria and antibody-based risk stratification is crucial for differentiating PNS from other n-irAEs and guiding appropriate management (15, 16). However, for cancer patients, conventional immunosuppressants such as tacrolimus, methotrexate, and cyclophosphamide should be used with caution to avoid accelerating tumor progression. Studies indicate that ICI therapy in patients with pre-existing PNS may exacerbate neurological symptoms and cause irreversible damage (17). Despite renewed academic interest in PNS following the incorporation of ICIs into oncologic practice, current understanding of ICI use in patients with PNS remains limited (18). Further exploration in this field is essential for guiding cancer treatment and preventing neurological dysfunction (19). This study analyzed the impact of paraneoplastic antibodies using 224 cases after PD-1/PD-L1 inhibitor–related PNS, assessing the effects of autoantibodies on neurological damage and prognosis, which might contribute to early risk stratification of n-irAEs and provide a theoretical basis for individualized immunomodulatory strategies.

## 2 Methods

This study was a retrospective descriptive study aimed at exploring the clinical and prognostic characteristics of PNS in cancer patients undergoing PD-1/PD-L1 inhibitor treatment. The study included both published literature data and cases from our hospital, and all cases met the contemporaneous diagnostic criteria for PNS, with a confirmed diagnosis. The study was approved by the Ethics Committee of the PLA General Hospital (HZKY-PJ-2022-22). As a non-interventional retrospective study, informed consent was waived, and all data were anonymized using coded identifiers.

A structured literature search was conducted in the PubMed, Embase, Wiley Online Library, and Web of Science databases from May 2015 to May 10, 2025. The search terms included “immune checkpoint inhibitors, ” “immunotherapy, ” “PD-1 inhibitor, ” “PD-L1 inhibitor, ” “neurological toxicity, ” “immune-related adverse events, ” “paraneoplastic neurological syndrome, ” “central nervous system, ” and “nervous system.

The inclusion criteria were as follows: (1) cancer patients with neurological adverse events that met the diagnostic criteria for PNS at the time of onset, referring to the 2004 criteria proposed by Graus et al. (20) and the updated July 2021 international consensus diagnostic framework (16); (2) publications providing detailed clinical data on neurological symptoms, diagnostic processes, and—when available—follow-up information.

A total of 216 patients from published literature were included. Each case was independently screened and data extracted by two researchers, with disputes resolved by a third investigator. Additionally, eight cases meeting the same criteria were retrieved from the neurology database of our hospital. All patients received anti–PD-1 or anti–PD-L1 monoclonal therapy between January 2018 and May 2025 and developed new or aggravated neurological symptoms during treatment, which were diagnosed as PNS after evaluation by a neurology specialist team.

**Figure 1:**
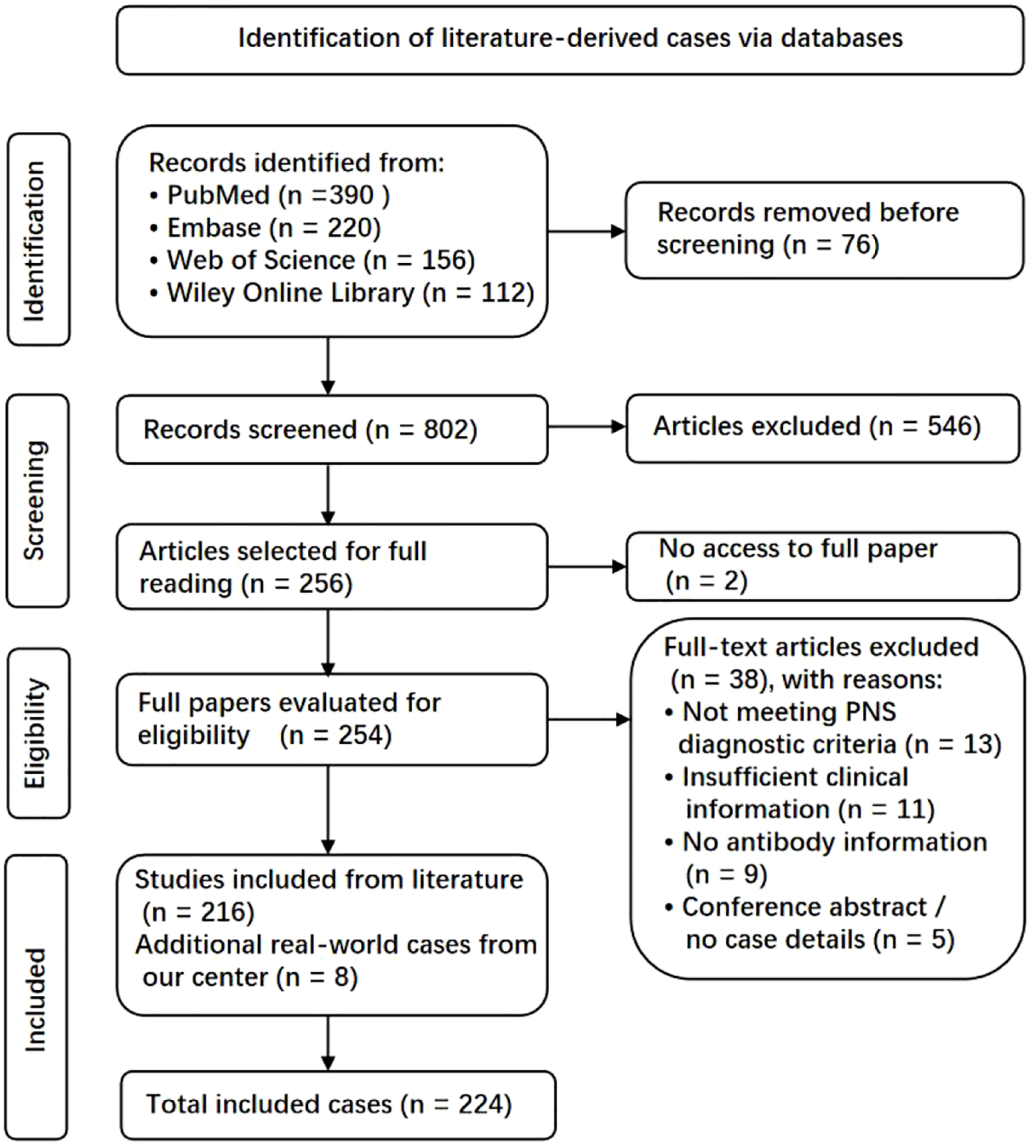
PRISMA flow diagram of case identification and selection. The figure illustrates the identification, screening, eligibility assessment, and final inclusion of literature-derived cases of PD-1/PD-L1 inhibitor–related paraneoplastic neurological syndromes, supplemented by additional real-world cases from our center.

The literature case identification process was conducted with reference to the PRISMA 2020 guidelines, solely to ensure transparent reporting of the screening process, and the study selection flow is presented in the PRISMA diagram (**Figure 1**).

Because the original reports spanned a wide publication period, PNS diagnoses had been established according to either the 2004 Graus criteria or the 2021 PNS-Care consensus framework. To ensure diagnostic consistency, we included only patients who fulfilled definite PNS under the 2004 criteria or probable/definite PNS according to the 2021 PNS-Care definitions. When sufficient clinical and antibody information was available, cases initially diagnosed using the 2004 criteria were re-evaluated for compatibility with the 2021 framework. Patients were then further stratified according to the 2021 PNS-Care risk-antibody classification into risk-antibody groups, unknown-risk antibody, and antibody-negative categories.

The neural autoantibodies analyzed in this study included those identified in our center’s cases as well as the tumor-related antibodies reported in the original publications, such as Anti-Hu, Anti-Ma and Anti-Ma2, Anti-Yo, Anti-amphiphysin, and neuronal-surface antibodies including Anti-NMDAR, and other related antibodies. For the eight patients from our center, serum and/or cerebrospinal fluid samples were examined in accredited neuroimmunology laboratories using standardized line-blot immunoassays and cell-based assays. For cases derived from the literature, antibody specificities were extracted directly from the source articles, most of which employed immunoblot-based or indirect immunofluorescence techniques comparable to those used in our center.

### 2.1 Data collection and outcomes

For all included patients, data were collected on age, sex, comorbidities, demographic characteristics, primary cancer types, immune therapy drugs used, clinical manifestations and subtypes of PNS, occurrence of immune-related adverse events, autoantibody test results, treatment strategies, treatment response, and clinical outcomes. For prognostic assessment, only cases with clearly documented final neurological outcomes were included. A favorable outcome was defined as a clearly described neurological improvement with recovery of a meaningful degree of functional independence. Any death reported during follow-up was classified as an unfavorable outcome. Cases without a clearly defined final neurological status were categorized as having an unknown prognosis and were excluded from the comparison between favorable and unfavorable outcomes.

Because the duration and method of follow-up were inconsistently reported across the original publications, we did not calculate a pooled mean or median follow-up time. Instead, we used the final neurological outcome as the primary endpoint, as this information was available for the vast majority of cases.

### 2.2 Statistical analysis

All data were subjected to descriptive statistical analysis. Categorical variables were expressed as frequencies and percentages, while continuous variables were presented as mean ± standard deviation or median (Q1, Q3), depending on data distribution. For group comparisons, Pearson’s Chi-square test was used for categorical variables, and Fisher’s exact test was applied when expected frequencies were <5. For continuous variables, one-way ANOVA was used when the data in each group met normal distribution and homogeneity of variance; if these assumptions were not satisfied, the Kruskal–Wallis test was used. A P-value <0.05 was considered statistically significant. All statistical analyses were performed using SPSS software (version 26.0, IBM Corporation, Chicago, USA).

## 3 Results

### 3.1 Baseline data and patient characteristics

A total of 224 cancer patients receiving PD-1/PD-L1 inhibitor treatment were included in this study, consisting of 112 in the risk-antibody group (87 high-risk, 20 intermediate-risk, and 5 low-risk), 51 in the unknown-risk antibody group, and 61 in the antibody-negative group. There were no significant differences in gender and age distribution among the three groups. Among all primary cancer types, lung cancer had the highest proportion, with 54%, 31%, and 48% in the risk-antibody, unknown-risk, and antibody-negative groups, respectively. The differences among the three groups were statistically significant (P < 0.001). Additionally, PD-1 inhibitors were commonly used in all three groups, and there was no significant difference in the time to the onset of neurological symptoms after the first PD-1/PD-L1 inhibitor treatment (**Table 1**), suggesting that the antibody status had no significant impact on the timing of symptom onset.

**Table 1:** Demographic and clinical characteristics of all cases, and whether autoantibodies to neuronal antigens were detected based on the patient group.

Characteristics	Total (n=224)	Risk-antibody (n=112)	Unknown-risk antibody (n=51)	Antibody negative (n=61)	P-value
Gender					0.872^a^
Male	134(60%)	67(60%)	30(59%)	34(56%)	
Female	90(40%)	45(40%)	21(41%)	27(44%)	
Age	63.5(53, 70)	63(53, 70)	63 (50, 75)	60.5 (45, 74)	0.628^b^
Cancer Type					< 0.001^c^
Lung Cancer	107(48%)	60(54%)	16(31%)	29(48%)	
Melanoma	29(13%)	4(4%)	15(30%)	10(16%)	
Other	88(39%)	47(42%)	20(39%)	22(36%)	
Metastasis	45(20%)	17(15%)	12(24%)	16(26%)	0.175^a^
ICI					0.008^a^
PD-1	159(71%)	75(67%)	26(51%)	48(79%)	
PD-L1	65(29%)	37(33%)	25(49%)	13(21%)	
Time of First ICI Treatment (weeks)	9(6, 14)	9(6, 15)	8(4, 11)	8.5(4, 13.3)	0.631^b^
Treatment					0.187^a^
Cytokine	64(29%)	35(31%)	15(29%)	14(22%)	
Blood Transfusion	28(13%)	12(11%)	7(14%)	9(15%)	
Monoclonal Antibodies	66(30%)	40(36%)	11(22%)	15(25%)	
Biological Agents	34(15%)	16(14%)	7(14%)	11(18%)	
Other	32(14%)	9(8%)	11(22%)	12(20%)	
Prognosis					
Good	124(55%)	41(37%)	37(72%)	46(75%)	< 0.001^a^
Death	46(21%)	32(29%)	8(17%)	6(10%)	0.012^a^

a: Pearson’s Chi-square Test; b: Kruskal-Wallis Test; c: Fisher’s Exact Test.

### 3.2 Impact of antibody status on patient prognosis

Among the different classifications of PNS, the most common diseases in the central nervous system across the risk-antibody, unknown-risk antibody, and antibody-negative groups included encephalomyelitis, subacute cerebellar degeneration, limbic encephalitis, and brainstem encephalitis. In the peripheral nervous system, acute sensory-motor neuropathy, subacute sensory neuronopathy, and autonomic neuropathy were most commonly observed. Among neuromuscular junction diseases, Lambert-Eaton myasthenic syndrome was the most frequent. Dermatomyositis was rare, with only two cases observed in our study. The incidence of limbic encephalitis, subacute cerebellar degeneration, and subacute sensory neuronopathy showed statistically significant differences among the risk-antibody, unknown-risk antibody, and antibody-negative groups (**Table 2**). Further analysis showed that the mortality rate was 29% in the risk-antibody group, compared with 17% in the unknown-risk antibody group and 10% in the antibody-negative group, and the differences between groups were statistically significant (P = 0.012) (**Table 1**).

**Table 2:** Distribution of PNS (paraneoplastic neurological syndrome) cases.

Diagnostic category	Total (n=224)	Risk-antibody (n=112)	Unknown-risk antibody (n=51)	Antibody negative (n=61)	P-value		
Central nervous system							
Encephalomyelitis	69(30.8%)	28 (25.0%)	19 (37.3%)	22 (36.1%)	0.401^a^
Limbic Encephalitis	35(15.6%)	23 (20.5%)	10 (19.6%)	2 (3.3%)	< 0.001^a^
Brainstem Encephalitis	9(4.0%)	4 (3.6%)	3 (5.9%)	2 (3.3%)	0.717^b^
Subacute Cerebellar Degeneration	52(23.2%)	31 (27.7%)	6 (11.8%)	15 (24.6%)	< 0.001^a^
Ocular Myoclonus - Muscle Spasms	1(0.4%)	0	0	1 (1.6%)	—
Stiff-Person Syndrome	1(0.4%)	0	1 (2.0%)	0 (0.0%)	—
Paraneoplastic Visual Impairment Syndrome	3(1.3%)	1 (0.9%)	1 (2.0%)	1 (1.6%)	1.000^b^
Paraneoplastic Optic Neuropathy	1(0.4%)	0	0	1 (1.6%)	—
Motor Neuron Disease	6(2.7%)	3 (2.7%)	0	3 (4.9%)	0.223^b^
Peripheral nervous system							
Subacute Sensory Neuronopathy	11(4.9%)	8 (7.1%)	0	3 (4.9%)	0.012^b^
Acute Sensory-Motor Neuropathy	16(7.1%)	7 (6.3%)	5 (9.8%)	4 (6.6%)	0.662^a^
Subacute Autonomic Neuropathy	5(2.2%)	2 (1.8%)	2 (3.9%)	1 (1.6%)	0.819^b^
Paraneoplastic Peripheral Neurovascular Inflammation	1(0.4%)	0	0	1 (1.6%)	—
Neuromuscular junction							
Lambert-Eaton Syndrome	11(4.9%)	4 (3.6%)	2 (3.9%)	5 (8.2%)	0.529^b^
Muscle							
Dermatomyositis	2(0.9%)	1 (0.9%)	1 (2.0%)	0	0.607^b^
Cachectic Myopathy	1(0.4%)	0	1 (2.0%)	0	—

a: Chi-Square Test; b: Fisher’s Exact Test.

### 3.3 Analysis of subtypes in the risk-antibody–positive patients

Among the 112 risk-antibody–positive patients, no statistically significant differences were found in terms of gender composition, types of PD-1/PD-L1 inhibitors used, and clinical prognosis (P > 0.05). However, the age distribution showed a statistically significant difference (P = 0.045) (**Table 3**). In our study, Anti-Hu antibody positivity was most commonly observed in limbic encephalitis, encephalomyelitis, subacute cerebellar degeneration, subacute sensory neuronopathy, and acute sensory-motor neuropathy. Additionally, a few cases of motor neuron syndrome and subacute autonomic neuropathy also showed Anti-Hu antibody positivity. Anti-Ma antibody positivity was more common in encephalomyelitis, limbic encephalitis, and subacute cerebellar degeneration. A small number of patients with paraneoplastic visual impairment, subacute sensory neuronopathy, and acute sensory-motor neuropathy also tested positive for Anti-Ma antibodies. Anti-Yo antibodies were primarily associated with subacute cerebellar degeneration, and three cases of encephalomyelitis also expressed Anti-Yo antibodies. Anti-amphiphysin antibodies were mainly associated with subacute cerebellar degeneration. Among the seven Anti-NMDAR antibody–positive patients included in our study, more than half presented with subacute cerebellar degeneration. Other types of positive antibodies were distributed across the central and peripheral nervous systems, as well as neuromuscular junction and muscle-related diseases. In addition, the incidence of limbic encephalitis differed significantly across the antibody groups (P = 0.005), with the highest incidence observed in the Anti-Hu antibody–positive group (**Table 4**).

**Table 3:** Characteristics of different types of neuronal antibodies.

Characteristic	Anti-Hu (n=43)	Anti-Ma (n=23)	Anti-Yo (n=12)	Anti-amphiphysin (n=6)	NMDAR (n=7)	Others (n=21)	P-value
Gender							0.313^a^
Male	28(65%)	16(70%)	5(42%)	4(67%)	2(29%)	12(57%)	
Female	15(35%)	7(30%)	7(58%)	2(33%)	5(71%)	9(43%)	
Age	61.8 ± 10.7	63.4 ± 12.6	60.8 ± 10.3	64.2 ± 7.8	58.6 ± 10.2	59.2 ± 12.9	0.045^b^
ICI treatment							0.348^a^
PD-1	28(65%)	18(78%)	5(42%)	4(67%)	6(86%)	14(67%)	
PD-L1	15(35%)	5(22%)	7(58%)	2(33%)	1(14%)	7(33%)	
Prognosis							
Good	16(37%)	8(35%)	4(33%)	1(17%)	5(71%)	7(33%)	0.491^a^
Death	10(23%)	8(35%)	3(25%)	2(33%)	2(29%)	5(24%)	0.915^a^

a: Fisher’s Exact Test; b: ANOVA.

**Table 4:** Distribution of PNS in different types of neuronal antibodies.

Diagnostic category	Total (n=112)	Anti-Hu (n=43)	Anti-Ma (n=23)	Anti-Yo (n=12)	Anti-amphiphysin (n=6)	NMDAR (n=7)	Others (n=21)	P-value								
Central nervous system																
Encephalomyelitis	28 (25.0%)	8 (18.6%)	11 (47.8%)	3 (25.0%)	0	4 (57.1%)	2 (9.5%)	0.089
Limbic Encephalitis	23 (20.5%)	15 (34.9%)	5 (21.7%)	0	1 (16.7%)	1 (14.3%)	1 (4.8%)	0.005
Brainstem Encephalitis	4 (3.5%)	0	0	0	0	1 (14.3%)	3 (14.3%)	0.625
Subacute Cerebellar Degeneration	31 (27.7%)	7 (16.3%)	4 (17.4%)	9 (75.0%)	4 (66.7%)	1 (14.3%)	6 (28.6%)	0.519
Paraneoplastic Visual Impairment Syndrome	1 (0.9%)	0	1 (4.3%)	0	0	0	0	—
Motor Neuron Disease	3 (2.7%)	2 (4.7%)	0	0	0	0	1 (4.8%)	1.000
Peripheral nervous system																
Subacute Sensory Neuronopathy	8 (7.1%)	5 (11.6%)	1 (4.3%)	0	0	0	2 (9.5%)	0.259
Acute Sensory-Motor Neuropathy	7 (6.3%)	4 (9.3%)	1 (4.3%)	0	0	0	2 (9.5%)	0.640
Subacute Autonomic Neuropathy	2 (1.8%)	2 (4.7%)	0	0	0	0	0	—
Neuromuscular junction																
Lambert-Eaton Myasthenic Syndrome	4 (3.6%)	0	0	0	1 (16.7%)	0	3 (14.3%)	0.625
Muscle																
Dermatomyositis	1 (0.9%)	0	0	0	0	0	1 (4.8%)	—

Fisher’s Exact Test.

**Figure 2:**
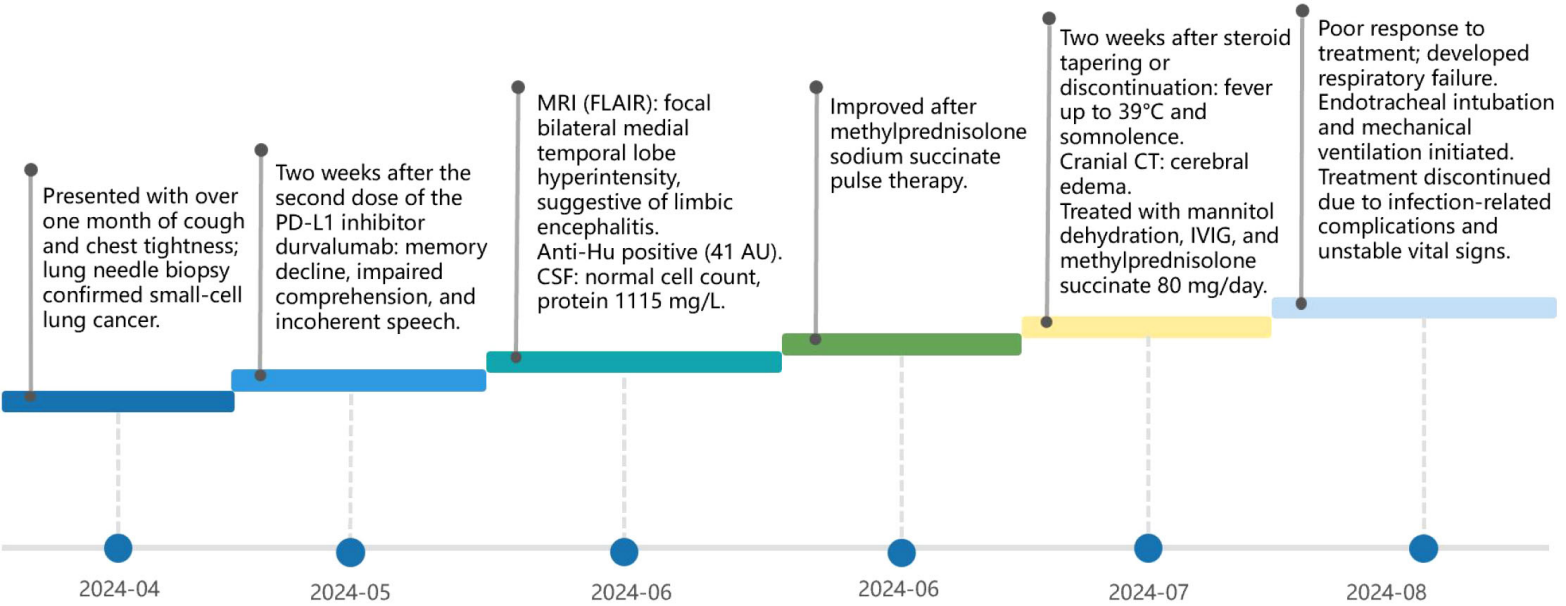
Clinical timeline of a patient with PD-L1 inhibitor durvalumab–related high-risk paraneoplastic neurological syndrome, anti-Hu–positive limbic encephalitis.

In terms of prognosis, the mortality rate was 23% in Anti-Hu–positive patients, 35% in Anti-Ma–positive patients, 25% in Anti-Yo–positive patients, and 33% in Anti-amphiphysin–positive patients. In contrast, 71% of Anti-NMDAR–positive patients had favorable outcomes. Although this difference did not reach statistical significance (P = 0.500), the results suggest that specific antibody types may be potentially associated with disease progression and treatment response (**Table 3**). To provide a clearer clinical context, a representative timeline from one of our center’s high-risk antibody–positive patients, who developed Anti-Hu–positive limbic encephalitis following PD-L1 inhibitor durvalumab therapy, is shown in **Figure 2**.

## 4 Discussion

Although n-irAEs are relatively rare among ICI-related toxicities, they are often severe and there are still no unified, evidence-based guidelines for their diagnosis and management (21, 22). From an immunopathogenic perspective, n-irAEs encompass a broad spectrum of off-target or off-tumor immune toxicities that may occur with or without identifiable neural autoantibodies, whereas PNS are driven by immune responses against onconeural antigens shared by the tumor and the nervous system (23, 24). Classical “spontaneous” PNS typically arise in patients with cancer who have never received ICIs, while PD-1/PD-L1 inhibitor–related PNS (PNS-ICI) represent either *de novo* induction or unmasking/exacerbation of pre-existing paraneoplastic autoimmunity under checkpoint blockade (23–26). Recent work shows that antibody-positive PNS-ICI share autoantibody profiles with spontaneous PNS but differ from other n-irAEs in tumor associations, clinical course, and implications for ICI continuation (23, 25, 26). Therefore, in this study we specifically focused on PD-1/PD-L1 inhibitor–related PNS, systematically evaluating their clinical characteristics and antibody profiles and analyzing their impact on prognosis, with the aim of providing a basis for early risk stratification and individualized intervention strategies.

In our cohort of 224 PD-1/PD-L1 inhibitor–related PNS cases, central nervous system presentations—such as encephalomyelitis, limbic encephalitis, and subacute cerebellar degeneration—were more frequent than peripheral neuropathies, neuromuscular junction disorders, or myopathies. At first sight, this pattern seems to differ from several large studies of neurological or peripheral nervous system immune-related adverse events after immune checkpoint inhibition, where inflammatory neuropathies and polyradiculoneuropathies have been described as the most common complications (27–30). The apparent inconsistency is largely attributable to differences in the patient populations examined. Those studies evaluated broad n-irAE cohorts without distinguishing paraneoplastic mechanisms, or they focused specifically on peripheral nervous system toxicities. In contrast, our study included only cases fulfilling contemporary diagnostic criteria for paraneoplastic neurological syndromes in the context of PD-1/PD-L1 blockade. Such antibody-mediated paraneoplastic autoimmunity is well known to present predominantly with central nervous system syndromes, particularly limbic encephalitis, encephalomyelitis and rapidly progressive cerebellar ataxia (31, 32). Aligned with this phenotype distribution, most antibody-positive patients in our series carried classical onconeural antibodies—including anti-Hu, anti-Ma/Ma2, anti-Yo and anti-amphiphysin—while neuronal-surface antibodies such as anti-NMDAR were uncommon. These differences in case definition and antibody profiles offer a coherent explanation for why central nervous system involvement predominated in our PD-1/PD-L1 inhibitor–related PNS cohort, even though peripheral neuropathy is more prominent in the broader n-irAE literature.

Recent work in neuroimmunology has shown that the interpretation of certain neurological presentations becomes more reliable when the clinical pattern is examined together with the available antibody information. In our cohort, limbic encephalitis, encephalomyelitis, rapidly progressive cerebellar ataxia and some forms of sensory neuronopathy or polyradiculoneuropathy were the most representative conditions. New studies have refined the description of these syndromes. Malvaso and colleagues reported that patients with LGI1- or CASPR2-associated limbic encephalitis may present with isolated disturbances of memory, particularly marked retrograde amnesia, and that cognitive recovery in such cases is often incomplete. Their findings suggest that selective memory impairment can be an early indication of limbic involvement (33). Research on neurological complications emerging during treatment with immune checkpoint inhibitors also points to a broad clinical spectrum. Zammit and colleagues summarized cases of limbic encephalitis, encephalomyelitis and polyradiculoneuropathy that showed considerable variability in antibody status, including seronegative forms. More recent observations from Lashkajani and others described central and peripheral nervous system involvement in patients receiving PD-1 or PD-L1 inhibitors (34, 35). These data are consistent with our findings. Clinical patterns regarded as having higher diagnostic significance tended to appear more frequently in the high- and intermediate-risk antibody groups, but were not limited to them, as similar presentations occurred in patients with uncertain or negative antibody results. This underlines the importance of assessing both the clinical syndrome and the antibody profile when evaluating possible paraneoplastic neurological complications during PD-1 or PD-L1 inhibitor therapy.

Of note, studies had indicated that the presence of paraneoplastic neural antibodies prior to ICI treatment was a risk factor for the development of PNS, with existing paraneoplastic neuropathy deteriorating after PD-1/PD-L1 inhibitor treatment (36, 37). Cross-reactive immune responses might occur when tumor cells abnormally expressed neural proteins, leading to immune system misjudgment (38), thereby exacerbating symptoms. Furthermore, prior experimental data showed that ICIs could induce PNS-like phenotypes in mice (39), and clinical reports indicated that patients might develop new paraneoplastic neurological syndromes or experience worsening of pre-existing PNS after ICI treatment (40). This phenomenon suggested an overlap between PNS and n-irAEs. Currently, the relationship between autoimmune-related antibodies, ICI treatment, and neurological malignancy remains incompletely understood (41). Relevant literature has reported potential associations, with Anti-Ma2–positive PNS patients experiencing worsening after ICI treatment (42). Moreover, Anti-Hu antibodies can react with nuclear antigens expressed in the brain, spinal cord, enteric neurons, and dorsal root ganglia (43). Therefore, synergistic pathogenic mechanisms between these antibodies and the primary disease may contribute to disease progression and poor prognosis. At present, guidelines do not recommend routine screening for PNS-related antibodies before initiating ICI therapy (16). In addition, based on the latest PNS diagnostic framework, some antibodies lack high specificity, and positive results may not reliably predict clinical events. According to our findings, it is advisable to evaluate PNS-related antibodies prior to PD-1/PD-L1 inhibitor treatment in the context of tumor characteristics, which is particularly important for reducing ICI-related neurotoxicity. Antibodies such as Anti-Hu, Anti-Ma2, or Anti-Yo may indicate more severe clinical manifestations and/or specific pathophysiological mechanisms (44, 45).

In our study, risk-antibody–positive patients had higher mortality than antibody-negative patients, while the prognosis of unknown-risk antibodies was similar to that of antibody-negative patients. Among the 112 cases in the risk-antibody group, high- and intermediate-risk antibodies predominated, with only five cases of low-risk antibodies. The 2021 PNS diagnostic criteria introduced the PNS-Care Score, which emphasized the diagnostic weight of high- and intermediate-risk antibodies and their causal relationship with malignancy (16). This further confirmed that the prognosis of PNS is worse than that of probable or possible PNS and underscored the necessity of antibody evaluation before initiating ICI therapy. As disease progresses, possible PNS without neuronal antibodies may later become antibody-positive, potentially expanding the antibody spectrum included in current risk stratification; this, however, requires confirmation in larger cohorts with long-term follow-up. We speculate that unknown-risk antibodies are not directly pathogenic and may induce weaker immune responses, resulting in less severe neurological injury.

In our study, the affected neural regions associated with certain antibody subtypes did not always align with common sites. For example, Anti-Hu antibodies were often closely associated with sensory neuropathy, cerebellar ataxia, and limbic encephalitis (46), whereas in our study, we observed that they could also cause acute sensory-motor neuropathy and subacute autonomic neuropathy. Additionally, Anti-Yo, Anti-Ma, and Anti-amphiphysin antibodies also presented with paraneoplastic visual impairment syndrome, subacute sensory neuronopathy, acute sensory-motor neuropathy, and Lambert-Eaton myasthenic syndrome, reflecting a diverse range of clinical manifestations. These rare forms of neurological injury might stem from immune system dysregulation following PD-1/PD-L1 inhibitor treatment. These findings underscored the long-term clinical challenges of managing cancer patients using PD-1/PD-L1 inhibitors, and the specific mechanisms required further exploration.

There were several limitations to this study: its retrospective design might have been influenced by data completeness and case selection bias. Because of the rarity of the disease, the literature search period was extended, which led to the coexistence of two diagnostic frameworks (2004 and 2021). Moreover, in many pre-2021 cases, follow-up outcomes were unavailable, making it difficult to assess longitudinal antibody changes and to derive a uniform follow-up duration for the overall cohort. Additionally, the lack of baseline serum samples from patients before receiving ICI treatment limited further analysis of antibody dynamic changes and causal relationships. Although this study included literature data and real-world cases from a single center, the overall sample size remained limited, and there might be referral bias toward more severe cases, potentially affecting the representativeness of disease spectrum distributions. Despite these limitations, this study provided a systematic presentation of the clinical characteristics of PD-1/PD-L1 inhibitor–related PNS, offering important insights for identifying high-risk populations and developing intervention strategies. Future studies should reclassify all available cases uniformly using the updated PNS diagnostic criteria, apply the PNS-Care Score for stratification, and conduct long-term follow-up to monitor antibody titers and subtypes over time, thereby enabling more precise investigations.

## 5 Conclusion

This study found that PD-1/PD-L1 inhibitor–related PNS predominantly involved the central nervous system, with risk-antibody–positive patients generally having worse prognoses. Significant differences in disease type and prognosis were observed between different antibody subtypes, suggesting that antibody status could serve as an important indicator for early identification of high-risk patients. Pre-treatment screening for neural antibodies may help to more precisely assess the neurotoxicity risk associated with ICI therapy and guide individualized intervention strategies.

## Data Availability

The raw data supporting the conclusions of this article will be made available by the authors, without undue reservation.
